# Long-Term Durability of Basalt Fiber-Reinforced Polymer (BFRP) Sheets and the Epoxy Resin Matrix under a Wet–Dry Cyclic Condition in a Chloride-Containing Environment

**DOI:** 10.3390/polym9120652

**Published:** 2017-11-28

**Authors:** Zhongyu Lu, Jianhe Xie, Huan Zhang, Jianglin Li

**Affiliations:** School of Civil and Transportation Engineering, Guangdong University of Technology, Guangzhou 510006, China; luzy@gdut.edu.cn (Z.L.); gdutzhanghuan@163.com (H.Z.); gdutlijianglin@163.com (J.L.)

**Keywords:** BFRP sheets, epoxy resin matrix, chloride ions, wet–dry cycle, residual tensile properties

## Abstract

Basalt fiber-reinforced polymer (BFRP) composites are receiving increasing attention as they represent a low-cost green source of raw materials. FRP composites have to face harsh environments, such as chloride ions in coastal marine environments or cold regions with salt deicing. The resistance of FRPs subjected to the above environments is critical for the safe design and application of BFRP composites. In the present paper, the long-term durability of BFRP sheets and the epoxy resin matrix in a wet–dry cyclic environment containing chloride ions was studied. The specimens of the BFRP sheet and epoxy resin matrix were exposed to alternative conditions of 8-h immersion in 3.5% NaCl solution at 40 °C and 16-h drying at 25 °C and 60% relative humidity (RH). The specimens were removed from the exposure chamber at the end of the 180th, 270th and 360th cycles of exposure and were analyzed for degradation with tensile tests, scanning electron microscopy (SEM) and void volume fractions. It was found that the tensile modulus of the BFRP sheet increased by 3.4%, and the tensile strength and ultimate strain decreased by 45% and 65%, respectively, after the 360th cycle of exposure. For the epoxy resin matrix, the tensile strength, tensile modulus and ultimate strain decreased by 27.8%, 3.2% and 64.8% after the 360th cycle of exposure, respectively. The results indicated that the degradation of the BFRP sheet was dominated by the damage of the interface between the basalt fiber and epoxy resin matrix. In addition, salt precipitate accelerated the fiber–matrix interfacial debonding, and hydrolysis of the epoxy resin matrix resulted in many voids, which accelerated the degradation of the BFRP sheet.

## 1. Introduction

Fiber-reinforced polymers (FRPs) are increasingly being used for repair and rehabilitation of existing structures [1,2]. The growing use of FRPs for strengthening and retrofitting can be explained by their specific strength and specific stiffness, good fatigue properties, ease of handling, and primarily resistance to corrosion [3]. Chloride ions are a major cause of corrosion of steel in concrete, which governs the effective life of the structures and their maintenance costs [4]. The corrosion-resistant characteristic makes FRPs ideal for applications subjected to the chloride ion environment [5,6]. Due to the versatile applications of concrete structures, FRP-reinforced concrete members may often be exposed to wetting and drying cycles, deicing salts in cold climates, sea salts in hot–humid climates, and many other severe environments [7].

In those applications, the long-term durability of the FRP composites is often one of the major advantages over the conventional materials [8,9,10]; durability is a significant consideration, especially because of the requirements of long service life without frequent inspection and maintenance [3]. Unfortunately, the durability of FRP composites is more complex than corrosion of steel reinforcement, because degradation of the material could depend both on the resin matrix and fibers and on their interface bond behavior [7,8,11]. Many previous studies have been conducted on the performance degradation of FRP-reinforced structures in a chloride-containing environment [4,5,6,9].

In one such study, the durability of a glass FRP (GFRP)/carbon FRP (CFRP)-reinforced concrete beam subjected to salt attack immersion was examined; the chloride ions easily permeated into the epoxy resin through the existing voids and cracks without causing hydrolysis to the vinyl ester resin [9,12]. Wang et al. [13] found that the chloride ions accelerated the hydrolysis of the epoxy resin matrix, and cracks increased in the specimens. Deterioration of the mechanical behavior can be accelerated by the exposure temperatures; long-term immersion of GFRP/CFRP in saltwater was found to be more damaging to the interlaminar shear strength, but the tensile modulus showed nearly no change after the immersion [9,14]. The test results also indicated that continuous immersion resulted in greater degradation than exposure to wetting and drying cycling [4,5], but the flexural fatigue strength was decreased by the process of water absorption and re-drying [15].

Understanding the durability of FRPs and their reinforced structures in a chloride-containing environment is necessary for their expanded use. Manuel et al. [16] studied the effects of tidal-like cycles (aqueous solution of NaCl, 50 g/L) on the mechanical properties of GFRP and CFRP laminates in an accelerated conditioning (12 h at 20% RH, followed by 12 h at 90% RH, and the temperature was kept at 35 °C); the mechanical tests showed degradation due to damage of the matrices and fiber–matrix linkage or adhesion between the fibers and the matrix. Wei et al. [17] studied the degradation of basalt fiber and glass fiber/epoxy resin composites in seawater; the tensile and bending strength of the specimens showed a decreasing trend with immersion time, and the diffusion of chloride ions around the composite toward the inside of the material is the main reason for corrosive degradation. Hydrolysis reaction in the composite may break the molecular chain and reduce the curing degree of the cross-linked network, resulting in the performance degradation of the composite.

Besides CFRPs and GFRPs, new high-performance composites have been developed [18], among which basalt fiber composites possess particular promise [19,20]. Basalt fibers are manufactured from basalt rocks through a melting process at 1400 °C, and without any other additives with reduced cost [8,21]. Basalt fibers have greater failure strain than carbon fibers and better tensile strength than E-glass fibers. Based on these advantages, the applicability of BFRPs for structural materials is highly expected [5,22,23]. Wu et al. [19] studied the durability of basalt fibers and composites in water, salt, acid and alkaline immersions. The resistance of the BFRP composite was much better than basalt fiber in those immersion tests, and the damage mechanism can be attributed to the chemical change in the acid solution and etching in the other solutions. Quagliarini et al. [24] and Lu et al. [8] claimed that the alkaline solution seriously affects the durability of the BFRP. Wang et al. [9] studied the durability of BFRP bars under seawater and sea sand concrete conditions; the degradation involved hydrolysis of resin and interface debonding. The degradation mechanism is also reported in the literature [8,21].

As mentioned previously, the existing literature provides only a limited understanding of the durability of BFRP composites under the wet–dry cyclic chloride-containing environment. Because of the variation in exposure conditions and different material parameters, it is difficult to compare these different test results and draw general conclusions. As such, this study examines the durability of BFRP sheets and the epoxy resin matrix under a wet–dry cyclic condition in a chloride-containing environment. The residual tensile properties of the exposed BFRP sheets and epoxy resin matrix were determined after the designed cycle times, one of which can be used to assess the durability of the BFRP sheet under a wet–dry exposure in a chloride-containing environment.

## 2. Experimental Program

### 2.1. Raw Materials

The unidirectional BFRP sheets (BUF13-4.5-380) used in this study were provided by the Shijin Basalt Fiber Co., Ltd. (Hangzhou, China). The weight of the basalt fiber sheets was 380 g/m^2^, the average diameter was 13 µm, the density was 1.796 g/cm^3^, and the nominal thickness was 0.167 mm. The tensile strength, tensile modulus and elongation at break were 1700 MPa, 84 GPa and 2.6%, respectively. The parameters were provided by the manufacturer.

The epoxy resin (HM-E8) used in this study was provided by Heroman chemical products Co., Ltd. (Guangzhou, China). It was a two-component resin with thixotropic epoxy adhesive and epoxy resin binder mixed in the ratio of 2:1 by weight, and the density was 1.6 g/cm^3^.

### 2.2. Preparation of the Specimens

The BFRP sheets were prepared in accordance with the guideline ASTM D 3039/D 3039 M-00 [25]. The specimen manufacturing process comprised two steps: The first step was to cut the BFRP sheet with the size of 250 × 25 × 0.167 mm. The second step was to impregnate the epoxy resin matrix on a piece of glass board, and a roller was used to remove any trapped air from within the specimens. The specimens were covered by another piece of glass board with something heavy, and cured at ambient room temperature for at least 7 days.

**Figure 1:**
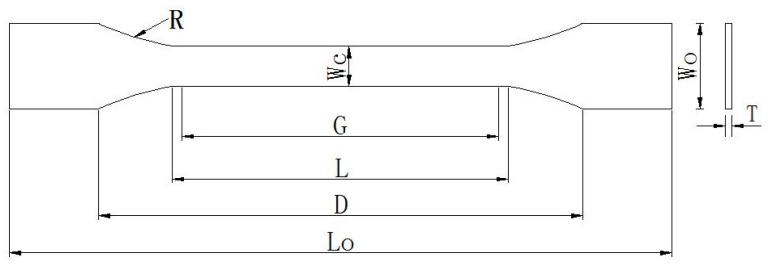
Schematic diagram of tensile specimens for the epoxy resin, *R*: radius of fillet; *L_O_*: length overall; *W_O_*: width overall; *D*: distance between grips; *L*: length of narrow section; *G*: gauge length; *W_C_*: width of narrow section; *T*: thickness.

The dog-bone-shaped resin specimens for the uniaxial tensile test were prepared following ASTM D 638-99 [26]. The epoxy resin matrix was mixed in the recommended proportion and transferred into an ultrasonic cleaning machine for 10 min with a 40 °C water environment. The epoxy resin matrix was then poured into the mold and tapped several times to remove any bubbles from the specimens. The specimens were cured at ambient room temperature for more than 7 days before being subjected to the exposure environment. The schematic diagram of the tensile specimens is shown in Figure 1, and the details are listed in Table 1.

### 2.3. Tensile Property Tests

**Figure 2:**
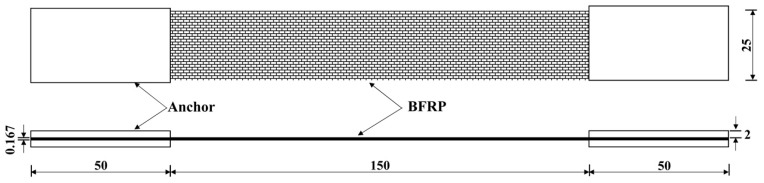
Basalt fiber-reinforced polymer (BFRP) specimens for tensile test.

**Figure 3:**
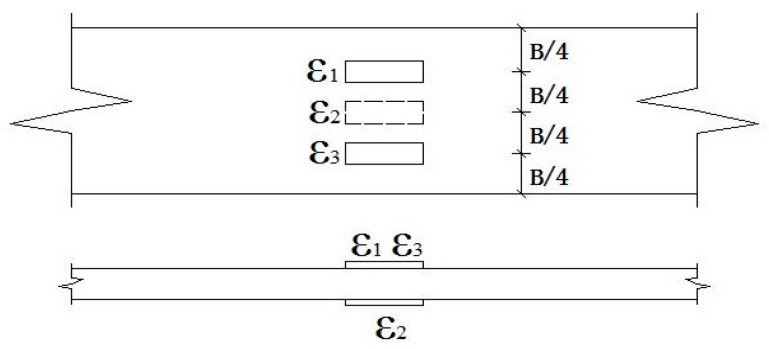
Strain gauge distributions of the BFRP sheet.

The BFRP specimens were prepared according to ASTM D 3039/D 3039 M-00 [25]. Aluminum sheets were used to anchor at both sides of these specimens, and the specimen details are shown in Figure 2. Tensile tests were carried out at a loading rate of 0.08 mm/min. The load was controlled by an electronic universal testing machine (DDL-300 model, Changchun Research Institute for Mechanical Science Co. Ltd., Changchun, China) equipped with a 300 kN load cell. The longitudinal strain of BFRP sheets was measured to determine the elastic modulus. The strain gauges (measurement type BHF 350-3AA) were provided by Zhejiang Huangyan Testing Apparatus Factory (Taizhou, China) and α-cyanoacrylate adhesive super glue (502 glue, purchased from a supermarket in Guangzhou, China) was used. The strain gauge distributions are illustrated in Figure 3. A TDS-530 high-speed static data logger (Tokyo Sokki Kenkyujo Co., Ltd., Tokyo, Japan) was utilized to record the data of the strain.

The strain can be obtained by the following formula:

(1) εa=(ε1+ε3)2+ε22,

where *ε*_1_, *ε*_2_ and *ε*_3_ are the strains obtained from the test. In order to ascertain the reliability and accuracy of the test results, the change in in-plane bending (*D*_1_) and out-of-plane bending (*D*_2_) were guaranteed within 5%. The expression of *D*_1_ and *D*_2_ are given as:

(2) $$D_{1}=\left|\frac{\varepsilon_{1}-\varepsilon_{3}}{\varepsilon_{a}}\right|\times 100\%,$$

(3) $$D_{2}=\left|\frac{\varepsilon_{a}-\varepsilon_{2}}{\varepsilon_{a}}\right|\times 100\%.$$

**Figure 4:**
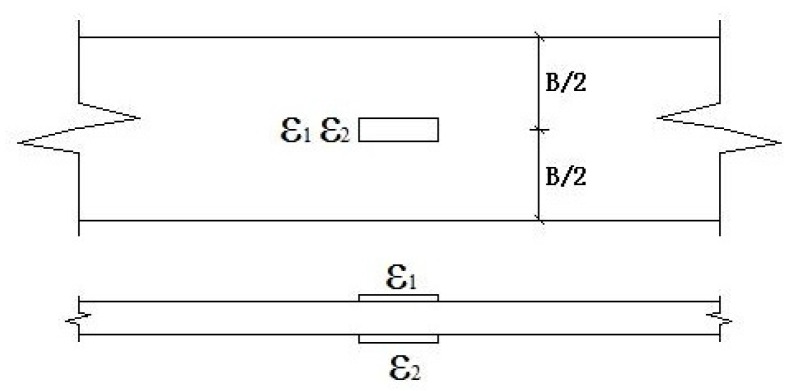
Strain gauge distributions of the epoxy resin matrix.

The tensile tests of the epoxy resin matrix were performed following ASTM D 638-99 [26]. The test instruments and parameters were the same as the BFRP sheet, and the only difference was the distribution of the strain gauges. As shown in Figure 4, two strain gauges were used in the test, and the strain was reported by the average value between the two.

The residual tensile property was performed at the end of the 180th, 270th and 360th cycles of exposure. At least three specimens were used for each test, and the average values of these results were reported.

### 2.4. Wet–Dry Cyclic Condition

**Figure 5:**
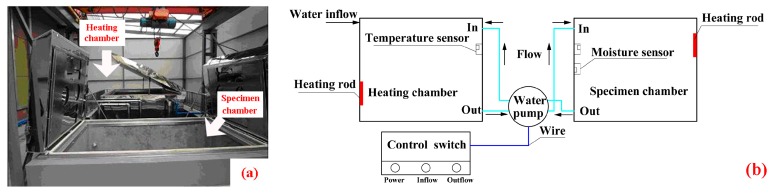
Wet–dry cyclic system, (**a**) physical map; (**b**) sketch map.

The wet–dry cyclic accelerated aging was performed at the structural laboratory, Guangdong University of Technology (Guangzhou, China), and the wet–dry cyclic system is shown in Figure 5. The concentration of the chloride ions (Cl^−^) was set as 3.5%. The process of a single wet–dry cycle was as follows: the specimens were immersed in 40 °C NaCl solution for 8 h, following by drying at 25 °C with 60% RH for 16 h. The temperature control precision was ±2 °C.

### 2.5. Scanning Electron Microscopy (SEM)

The surfaces of the BFRP sheet and the tensile fracture of the epoxy resin matrix were platinum coated for 10 min and observed using scanning electron microscopy (model S-3400N-II, Hitachi, Tokyo, Japan).

### 2.6. Void Volume Fractions Test

Based on the literature [27], the void volume fractions of the BFRP and resin matrix were calculated. The void volume fraction was calculated by the following equation:

(4) $$\varepsilon =\left(1-\frac{m}{Z\times A\times \rho }\right)\times 100\%,$$

where *ε* is the void volume fraction, and *m*, *Z* and *A* are mass, thickness and area per unit measure membrane, respectively. ρ is the density of the specimens. The mass of the specimens was tested using an electronic balance (model AR224CN) with an accuracy of ±0.1 mg, provided by Ohaus Instruments Co., Ltd. (Changzhou, China).

## 3. Results and Discussions

### 3.1. Tensile Strength

**Figure 6:**
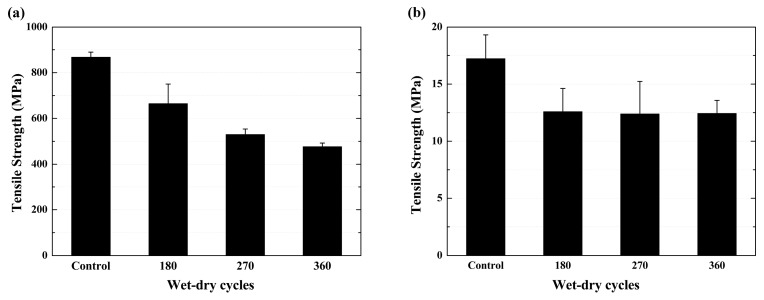
Tensile strength of the specimens as a function of wet–dry cycles, (**a**) BFRP sheet; (**b**) epoxy resin matrix.

Figure 6 shows the tensile strength of the specimens after various exposure cycles. As shown in Figure 6a, the tensile strength of the BFRP sheet decreased as the exposure cycle increased, and the tensile strength decreased by 23.4%, 38.9% and 45.1% (compared to its original value of 867.9 MPa) after 180, 270 and 360 cycles, respectively. The tensile strength of the epoxy resin matrix is shown in Figure 6b, and a much more severe decrease at the end of the 180th cycle of exposure can be found. The tensile strength decreased from 17.2 MPa to 12.6 MPa (decreased by 26.7%), and then kept almost constant as the exposure cycles increased. It is worth noting that the tensile strength of the epoxy resin matrix showed much more degradation at the end of the 180th cycle of exposure, and this tensile strength was exceeded by the BFRP sheet in the subsequent test. The tensile strength of a material is more closely related to the development of cracks in the materials, which is more sensitive to the defects present [7,9,20]. The degradation of tensile strength can also be related to the plasticization, which reduces the interaction between molecules. The increased exposure may also lead to relaxation of the stress concentration formed during curing. As a result, the tensile strength did not vary much as the number of cycles increased [10].

**Figure 7:**
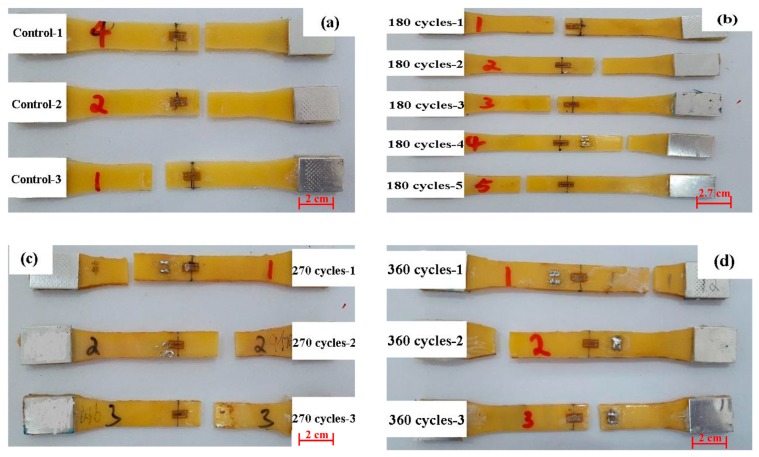
Failure modes of the epoxy resin matrix, (**a**) control; (**b**) 180 cycle times; (**c**) 270 cycle times; (**d**) 360 cycle times.

The properties of the FRP composite are governed not only by the fiber and the resin matrix, but also by the interface between the fiber and epoxy resin matrix [20]. Based on the above analysis, the degradation of the BFRP sheet may be induced by the damage of the basalt fiber or the interface between the basalt fiber and epoxy resin matrix. The chloride ions can easily permeate into the epoxy resin through the existing voids and cracks, and destroy the epoxy resin matrix [13]. Based on the test results of the BFRP specimen exposure for 180 cycles, the void volume fractions increased from 0.33 (control) to 0.41. Figure 7 shows the failure mode of the epoxy resin matrix specimens after various cycles. There is no clear difference of the failure modes between the specimens exposed to the accelerated aging environment or not. The specimens were fractured almost in the middle part with brittle failure mode. Moreover, the color deepened as the number of exposure cycles increased. This can be attributed to the high concentration of dissolved salts that permeated into the specimens; the salt precipitated during the wet–dry cycle [28], and the damage increased the void volume fractions of the epoxy resin. Compared with the original value, it increased from 0.19 to 0.41 as the exposure time increased to 180 cycles.

**Figure 8:**
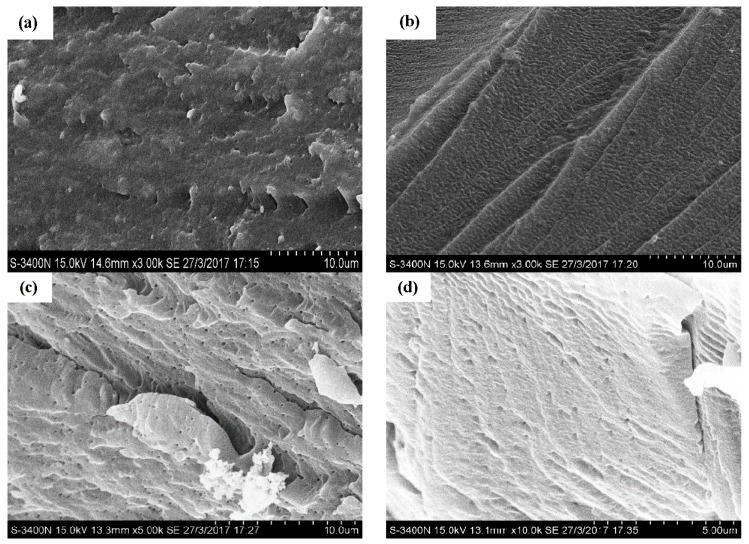
The morphologies of the epoxy resin matrix, (**a**) control; (**b**) 180 cycles; (**c**) 270 cycles; (**d**) 360 cycles.

Scanning electron microscopy (SEM) morphologies of the tensile fracture surface of the epoxy resin matrix are illustrated in Figure 8. For the control specimen (Figure 8a), no obvious cracks and voids were observed. As the exposure increased to 180 cycles, a few small cracks and wrinkles appeared due to the penetration of chloride ions, as shown in Figure 8b. That is the reason for the tensile strength deterioration of the epoxy resin matrix. For the specimens exposed after the 270th and 360th cycles of exposure (Figure 8c,d), obvious cracks and voids were noticed. The coalescence of voids and micro-cracks into macro-cracks was observed, which in turn provided new paths for diffusion and wicking of chloride ions [9,29]. Thus, the level of moisture transport was enhanced, and significantly affected the voids in the specimen. There is another reason for the deepened color of the specimen. For some unknown reason, a threshold was reached at the end of the 180th cycle of exposure, and the developed defects showed little effect on the tensile strength of the epoxy resin matrix.

**Figure 9:**
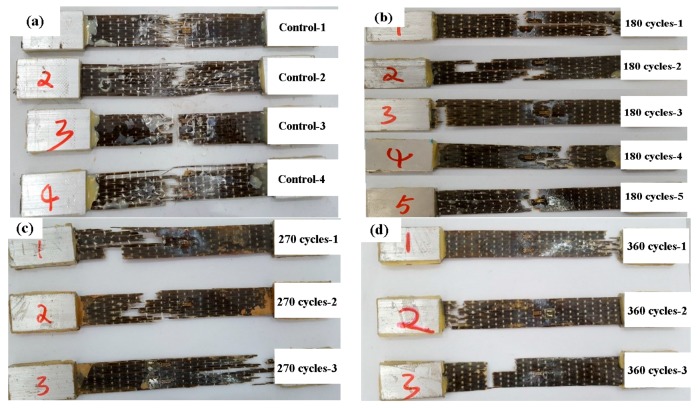
Failure modes of BFRP sheets, (**a**) control; (**b**) 180 cycles; (**c**) 270 cycles; (**d**) 360 cycles.

In addition to the epoxy resin matrix, basalt fiber and the interface between the basalt fiber and epoxy resin matrix can also lead to degradation of the performance of the BFRP sheet. Figure 9 shows photographs of the BFRP sheets after tensile tests at various exposure cycles. The control specimen is shown in Figure 9a; the specimens were fractured in the middle part with a visible zigzag failure mode. As the exposure increased to 180 cycles (Figure 9b), the splitting failure occurred in a brittle manner. This failure mode often began at one of the loaded edges with defects and propagated into the pane [20,28]. With the increase in the number of exposure cycles, the fractured cross-section became regular, as shown in Figure 9c,d for the specimens at the end of the 270th and 360th cycles of exposure, respectively. The failure model may be attributed to the degradation of the basalt fiber and the interface between the fiber and matrix [8]. Fe^2+^ exists in basalt fiber (about 10%) [30], and the chemical reactions involved are described as follows [17]:

(5) $${\mathrm{Fe}}^{2+}+{\mathrm{Cl}}^{-}\to {\left[\mathrm{FeCl}\;\mathrm{complex}\right]}^{-}.$$

Therefore, the reaction may explain the degradation of BFRP sheets under the exposure environment. As the relative content of ferrous ion is low, the interface damage between the basalt fiber and epoxy resin matrix is another reason for the performance degradation of the BFRP sheet. Water diffusion through the interface into the BFRP sheets [31] and the chloride ions accelerated the hydrolysis of the epoxy resin matrix [13]. Cracks, voids and deterioration of the interface between the basalt fiber and epoxy resin matrix occurred [9,32], and salt precipitation during the wet–dry cycle accelerated the development of cracks, followed by fiber–matrix interfacial debonding and coalescence of voids and micro-cracks into macro-cracks [29].

**Figure 10:**
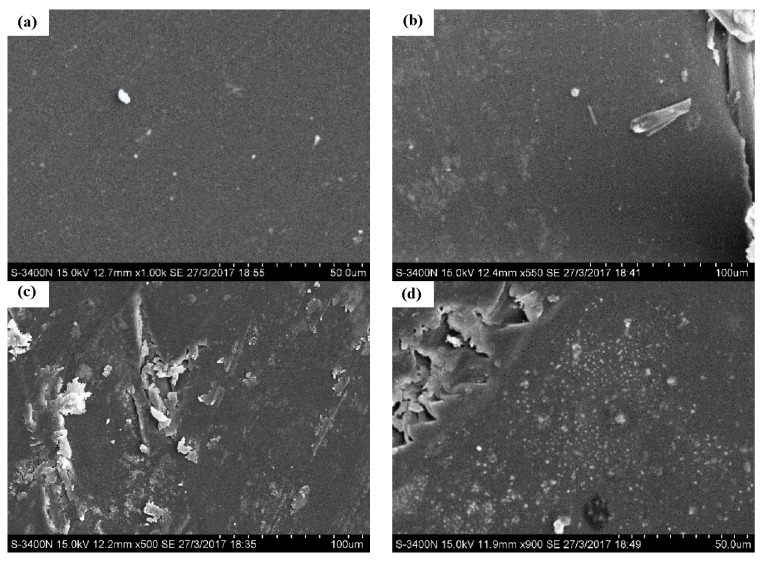
The morphologies of the BFRP sheets, (**a**) control; (**b**) 180 cycles; (**c**) 270 cycles; (**d**) 360 cycles.

The interface damage is illustrated in Figure 10 by scanning electron microscopy (SEM) photographs. For the control specimen, as shown in Figure 10a, a smooth surface can be found. As shown in Figure 10b, the surface morphology of the specimens after 180 cycles featured no obvious changes compared with the control specimen. Figure 10c shows the specimen at the end of the 270th cycle of exposure; serious damage can be observed on the surface. This can be attributed to the hydrolysis of the epoxy resin matrix [13]. More serious damage can be found for the specimen after 360 cycles of exposure (Figure 10d). The longer the exposure time, the more damage can be found. The damage of the epoxy resin matrix led to the interface damage, which contributed to the degradation of the BFRP sheet under the wet–dry cyclic condition in the chloride-containing environment.

**Figure 11:**
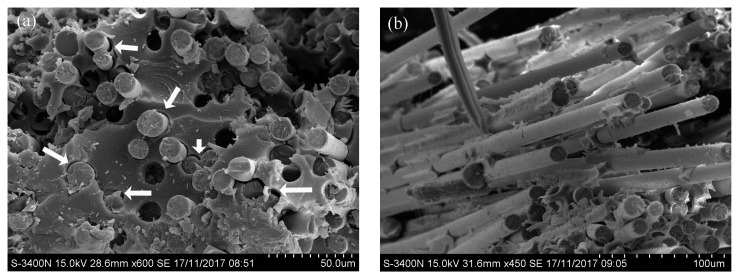
The morphologies of the BFRP sheets exposed for 180 cycles, (**a**) interface; (**b**) fiber.

The SEM test results of the specimens exposed for 180 cycles are shown in Figure 11. As can be seen in Figure 11a, basalt fibers were pulled out of the specimens, and gaps can be found between the basalt fiber and resin matrix. This phenomenon indicates the interface damage in the specimens. As for the basalt fibers, there was no damage found on the surface of the basalt fibers, as shown in Figure 11b.

### 3.2. Tensile Modulus

**Figure 12:**
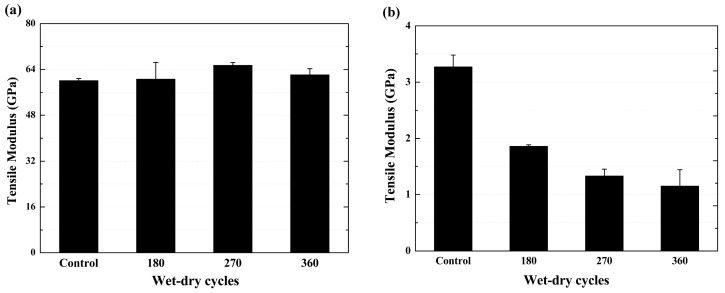
Tensile modulus of the specimens as a function of wet–dry cycles, (**a**) BFRP sheet; (**b**) epoxy resin matrix.

Figure 12 shows the tensile modulus of the specimens after various exposure cycles. As shown in Figure 12a, the tensile modulus of the BFRP sheet showed a slight increase as the exposure cycle increased. Compared to the original value of 60.1 GPa, the tensile modulus increased by 8.9% at the end of the 270th cycle of exposure, and then decreased to 62.1 GPa (increased by 3.4% compared to its original value). The tensile modulus is a measure of the deformation to external stress, which is determined by the interaction forces between atoms or molecules [20]. Thus, the retention of the tensile modulus is higher than the tensile strength of the BFRP sheet. Similar test results were reported on the higher retention of the tensile modulus of FRP composites, and a possible reason for this was the post cure of the specimen at the exposure temperatures [9,14,19,33,34]. An opposite trend appeared: The tensile modulus of the epoxy resin matrix (Figure 12b) decreased more compared to its tensile strength (see Figure 6b), even if the defects were developed. The tensile modulus of the epoxy resin matrix decreased by 43.1%, 59.3% and 64.8% (compared to its original value of 3.3 GPa) as the exposure cycles increased to 180, 270 and 360, respectively. The decreased tensile modulus can be attributed to the plasticizing of the epoxy resin matrix [1,5].

**Figure 13:**
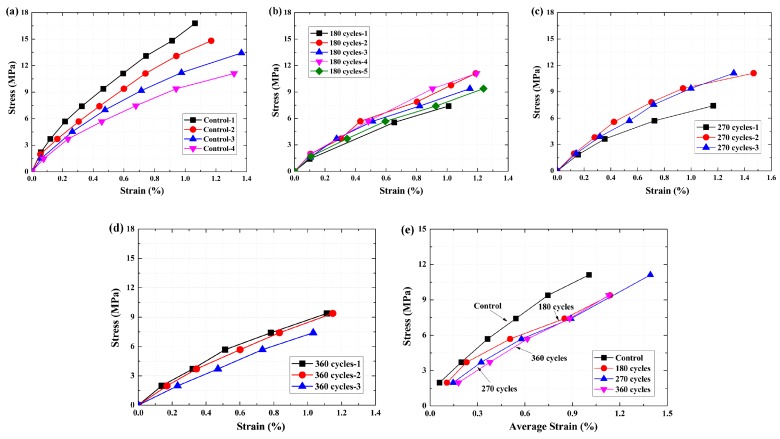
Mechanical properties of the epoxy resin matrix with increasing wet–dry cycles, (**a**) control; (**b**) 180 cycles; (**c**) 270 cycles; (**d**) 360 cycles; (**e**) average strain.

The stress–strain curves of the epoxy resin matrix are shown in Figure 13. As shown in Figure 13a–d, an almost linear relationship between the stress and strain can be found for all specimens. The maximum stress decreased, and the deformation increased at the same load level as the exposure cycles increased. Thus, the slope of these curves decreased, resulting in a decrease of the tensile modulus. To clearly show the effects of the exposure on the stress–strain relationship, the average of the stress–strain curves is shown in Figure 13e. Obvious difference can be found for the control specimen, and the slope of the curve decreased after 180 cycles. As the number of exposure cycles continued to increase, there was no obvious difference in the slope between the stress–strain curves. The results agree well with the change of the tensile strength and tensile modulus of the epoxy resin matrix.

**Figure 14:**
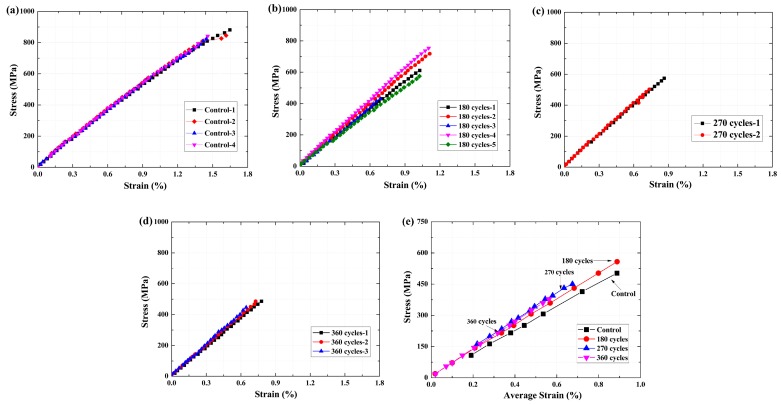
Mechanical properties of the BFRP sheets with increasing wet–dry cycles, (**a**) control; (**b**) 180 cycles; (**c**) 270 cycle cycles; (**d**) 360 cycle cycles; (**e**) average strain.

As a contrast, the stress–strain curves of the BFRP sheet are shown in Figure 14. A linear relationship can be found for all specimens after various exposure cycles. As shown in Figure 14a–d, the maximum stress decreased as the exposure cycles increased. The various slopes of these curves agree well with the change in the tensile modulus. To clearly show the effects of the exposure cycle, the average of the stress–strain curves is shown in Figure 14e. At the same load level, the deformation ability of the specimen increased as the number of exposure cycles increased. This may be the reason for the tensile modulus remaining almost constant, even if the defects developed and resulted in a decrease in the tensile strength. Compared with the epoxy resin matrix, the deformation of the BFRP sheet is smaller than that of the epoxy resin matrix at the same load level. The results indicated that the deformation was controlled by the basalt fiber, and the contribution of the epoxy resin matrix to the tensile strength of the BFRP sheet is limited [19,34]. Due to the unevenness of the basalt fiber in the BFRP sheet [8,22], and because the basalt fibers lack the protection of the epoxy resin matrix [20], corrosion of basalt fiber or the epoxy resin matrix occurred in this region [31]. Thus, the interfacial stress-transfer efficiency decreased, resulting in a decrease in the tensile strength of the BFRP sheet.

### 3.3. Ultimate Strain

**Figure 15:**
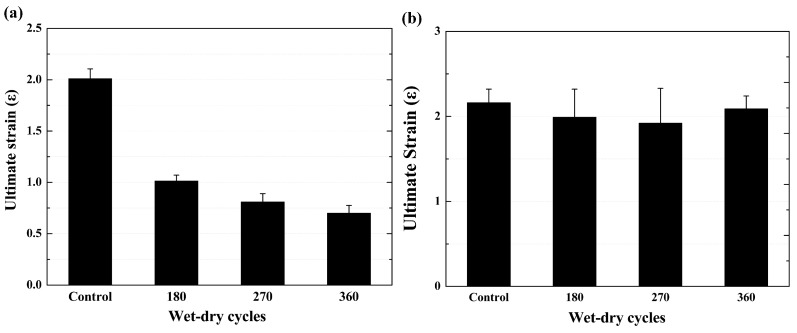
Ultimate strain of the specimens as a function of wet–dry cycles, (**a**) BFRP sheet; (**b**) epoxy resin matrix.

Figure 15 shows the ultimate strain of the specimens after various exposure cycles. As shown in Figure 15a, the ultimate strain of the BFRP sheet decreased as the number of exposure cycles increased, and the ultimate strain decreased by 52.3%, 61.9% and 66.7% (compared to its original value of 2.1%) at the end of the 180th, 270th and 360th cycles of exposure, respectively. Regarding the epoxy resin matrix (Figure 15b), compared with the original value of 2.2%, the ultimate strain decreased by 7.8%, 11.1% and 3.2% at the end of the 180th, 270th and 360th cycles of exposure, respectively. Plasticization is used to explain why the strain does not change much. Deepened color indicates the degradation of the resin on the surface of the specimen. The thickness is very limited, and will not affect the whole specimen [10]. The results also indicated that the deformation of the BFRP sheet is governed by the basalt fiber, and the interface damage occurred between the basalt fiber and epoxy resin matrix during the tensile test due to the different deformation abilities of the two components of the composite. Furthermore, the corrosion of the basalt fiber increased the brittleness of the composite [34]. Thus, the ultimate strain of the BFRP sheet decreased as the exposure cycles increased.

**Figure 16:**
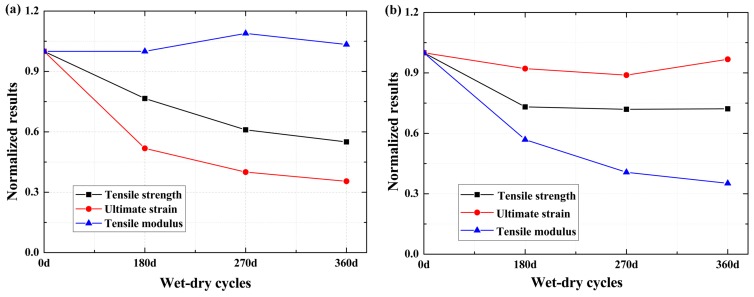
Mechanical properties of the specimens with an increasing number of wet–dry cycles, (**a**) BFRP sheets; (**b**) epoxy resin matrix.

To compare the performance degradation of the specimens under the effect of exposure, the normalized results of the specimens are shown in Figure 16. For the normalized results of the BFRP sheet, as shown in Figure 16a, the retention of the tensile modulus is the highest. This can be attributed to the tensile modulus being insensitive to the defects [7,9,20], and the deformation ability decreasing more compared with the decreases of the tensile strength. Thus, the retention of the ultimate strain is the lowest. The normalized results of the epoxy resin matrix are shown in Figure 16b; the retention of the ultimate strain is the highest. The ultimate strain of the epoxy resin matrix almost remained constant, which can be attributed to the plasticizing of the epoxy resin matrix that increased the deformation ability of the specimen [1,5]. The tensile strength of the epoxy resin decreased, and the slope of the stress–strain curve decreased. Thus, the retention of the tensile modulus is the lowest.

## 4. Conclusions

An experimental study has been conducted to investigate the long-term durability of the BFRP sheet and the epoxy resin matrix under a wet–dry cyclic condition in a chloride-containing environment. The residual tensile properties and void volume fractions were calculated, and SEM micrographs obtained. The following conclusions have been drawn from the experimental results and discussion:

1. The wet–dry cycles with chloride-containing solution caused the hydrolysis of the epoxy resin matrix, and interfacial damage between the basalt fiber and epoxy resin matrix. Salt precipitation accelerated the fiber–matrix interfacial debonding during the wet–dry cycles, resulting in serious degradation of the tensile strength of the BFRP sheet. The longer the exposure time, the more serious the degradation found.
2. Chloride ions accelerated the penetration and degradation of the epoxy resin matrix, resulting in the degradation of the tensile strength of the resin matrix. With the increase in the number of cycles, more water ingressed led to more plasticization, which was responsible for the reduction in the tensile modulus.
3. The deformation was governed by the basalt fiber for the BFRP sheet. The decreased stress-transfer at the interface between the basalt fiber and resin matrix led to degradation of the tensile strength of the BFRP sheet, but the tensile modulus remained approximately constant as the cycling time increased.

## Figures and Tables

**Table 1:** Details of the tensile specimens for the epoxy resin.

Characteristic	*R*—Radius of Fillet	*L_O_*—Length Overall	*W_O_*—Width Overall	*D*—Distance between Grips	*L*—Length of Narrow Section	*G*—Gauge Length	*W_C_*—Width of Narrow Section	*T*—Thickness
Dimension (mm)	76	165	19	115	57	50	13	3
